# Two Pex5 Proteins With Different Cargo Specificity Are Critical for Peroxisome Function in Ustilago maydis

**DOI:** 10.3389/fcell.2022.858084

**Published:** 2022-05-12

**Authors:** Julia Ast, Nils Bäcker, Elena Bittner, Domenica Martorana, Humda Ahmad, Michael Bölker, Johannes Freitag

**Affiliations:** ^1^ Department of Biology, Philipps-University Marburg, Marburg, Germany; ^2^ Institute of Metabolism and Systems Research (IMSR), and Centre of Membrane Proteins and Receptors (COMPARE), University of Birmingham, Birmingham, United Kingdom; ^3^ Center for Synthetic Microbiology, Philipps-University Marburg, Marburg, Germany

**Keywords:** PEX5, PEX7, beta oxidation, peroxisome, targeting signal, Ustilago maydis, PTS1, PTS2

## Abstract

Peroxisomes are dynamic multipurpose organelles with a major function in fatty acid oxidation and breakdown of hydrogen peroxide. Many proteins destined for the peroxisomal matrix contain a *C*-terminal peroxisomal targeting signal type 1 (PTS1), which is recognized by tetratricopeptide repeat (TPR) proteins of the Pex5 family. Various species express at least two different Pex5 proteins, but how this contributes to protein import and organelle function is not fully understood. Here, we analyzed truncated and chimeric variants of two Pex5 proteins, Pex5a and Pex5b, from the fungus *Ustilago maydis.* Both proteins are required for optimal growth on oleic acid-containing medium. The *N*-terminal domain (NTD) of Pex5b is critical for import of all investigated peroxisomal matrix proteins including PTS2 proteins and at least one protein without a canonical PTS. In contrast, the NTD of Pex5a is not sufficient for translocation of peroxisomal matrix proteins. In the presence of Pex5b, however, specific cargo can be imported *via* this domain of Pex5a. The TPR domains of Pex5a and Pex5b differ in their affinity to variations of the PTS1 motif and thus can mediate import of different subsets of matrix proteins. Together, our data reveal that *U. maydis* employs versatile targeting modules to control peroxisome function. These findings will promote our understanding of peroxisomal protein import also in other biological systems.

## Introduction

In eukaryotic cells specific metabolic pathways are often contained inside of organelles such as mitochondria and peroxisomes (Lodish et al., 2000). Peroxisomes have versatile biological roles including detoxification of hydrogen peroxide, degradation of fatty acids and metabolism of amino acids and are essential for human health (Smith and Aitchison, 2013; Wanders, 2014). Peroxisomal matrix proteins are imported into the organelle lumen from the cytosol *via* an evolutionary conserved set of cytosolic receptors and peroxisomal membrane proteins (Gabaldón, 2010; Kim and Hettema, 2015; Walter and Erdmann, 2019). The majority of known matrix proteins contains one of two conserved targeting signals termed peroxisomal targeting signal type 1 (PTS1) and type 2 (PTS2). PTS1 motifs are located at the *C*-terminus, originally identified as a tripeptide with the sequence Ser-Lys-Leu (SKL), occurring in many variations of this prototype sequence (Gould et al., 1989; Brocard and Hartig, 2006; Lingner et al., 2011; Nötzel et al., 2016). The amino acid sequence upstream of the *C*-terminal tripeptide contributes to PTS1 recognition (Brocard and Hartig, 2006). PTS2 motifs are located in the *N*-terminal part of a protein and are recognized by the receptor protein Pex7 (Braverman et al., 1997; Lazarow, 2006; Kunze et al., 2011; Kunze, 2020). Several proteins lacking canonical targeting signals have been described (van der Klei and Veenhuis, 2006). These either rely on piggy-back import mediated by their interaction with other PTS-containing proteins (Glover et al., 1994; McNew and Goodman, 1994; Islinger et al., 2009; Schueren et al., 2014; Effelsberg et al., 2015; Saryi et al., 2017; Gabay-Maskit et al., 2020) or on direct interaction with Pex5 (Skoneczny and Lazarow, 1998; Klein et al., 2002; Rymer et al., 2018; Kempiński et al., 2020; Rosenthal et al., 2020; Yifrach et al., 2021).

Pex5 recognizes PTS1 motifs *via* several tetratricopeptide repeats (TPRs) located in its *C*-terminal domain (CTD) (Brocard et al., 1994; Gatto et al., 2000). Subsequently, the receptor-cargo complex interacts with the peroxisomal membrane proteins Pex13 and Pex14 (Gould et al., 1996; Urquhart et al., 2000; Lill et al., 2020), followed by import of cargo proteins into the peroxisomal lumen without the requirement for ATP turnover (for review see: Miyata and Fujiki, 2005; Kim and Hettema, 2015; Francisco et al., 2017). The interaction with Pex14 is mediated *via* a conserved di-aromatic amino acid motif located within the unstructured *N*-terminal domain (NTD) of Pex5 (Saidowsky et al., 2001; Otera et al., 2002; Carvalho et al., 2006; Su et al., 2009). The exact mechanistic functionality of the translocation machinery is still a matter of investigation and so far lacks structural data, but probably has a transient character (Meinecke et al., 2010; Dias et al., 2017; Bürgi et al., 2021). After cargo release, Pex5 proteins are recycled from the peroxisome to the cytosol. Recycling involves ubiquitination, unfolding, energy provided by the AAA-ATPases Pex1 and Pex6, and deubiquitination (Miyata and Fujiki, 2005; Platta et al., 2005; Platta et al., 2007; Gardner et al., 2018; Pedrosa et al., 2018; El Magraoui et al., 2019).

In mammals, two isoforms of Pex5 – Pex5-small and Pex5-large – are generated that derive from alternative splicing (Braverman et al., 1998). The longer isoform contains an additional Pex7 binding domain inside of the NTD. In mammals, another TPR-containing protein with significant homology to Pex5 was identified, which can interact with PTS1 proteins but also with an ion channel (Amery et al., 2001; Santoro et al., 2004, 2011). Many fungi encode two proteins with homology to the PTS1 receptor Pex5 (Kiel et al., 2006; Freitag et al., 2012). In *Saccharomyces cerevisae*, the Pex5 paralog Pex9 is induced in cells grown in oleic acid-containing medium and controls peroxisomal import of the glyoxylate cycle enzyme malate synthase and additional cargo (Effelsberg et al., 2016; Yifrach et al., 2016; Yifrach et al., 2022). Thus, distinct pathways to target PTS1 proteins to peroxisomes are found in diverse eukaryotes and may be crucial for the regulation of peroxisomal protein import and peroxisome function.

**FIGURE 1:**
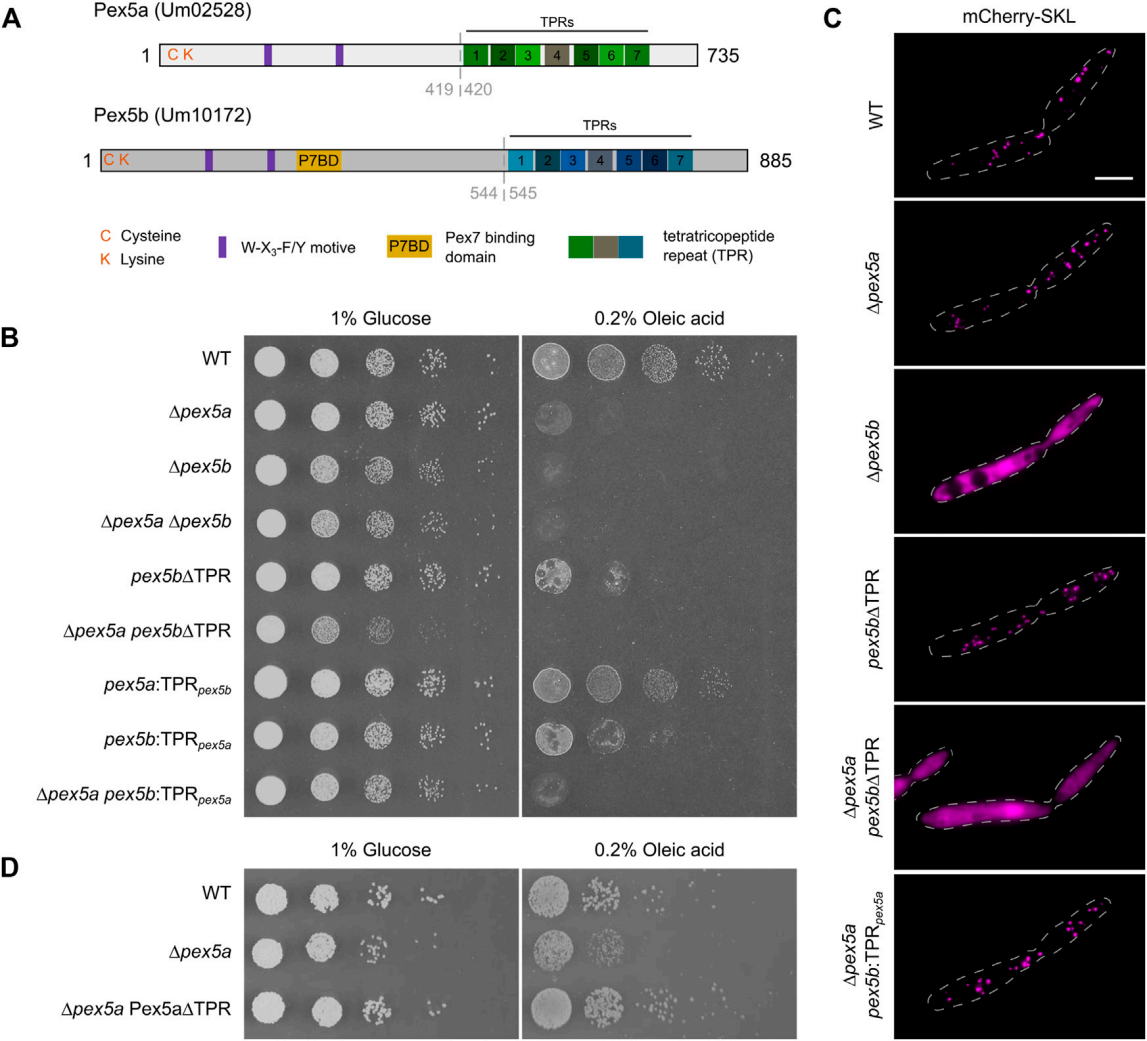
Contribution of the *N*- and *C*-terminal domains of two Pex5 proteins to peroxisome function. **(A)** Scheme illustrating the domain organization of Pex5a and Pex5b from *U. maydis*. C and K refer to a cysteine and a lysine residue possibly involved in ubiquitination. Purple rectangles denote the position of W-X_3_-F/Y motifs required for docking complex interaction. A putative Pex7 binding domain (P7BD) of Pex5b is shown in yellow. The position of the TPR domains is highlighted with rectangles. Gray dashed lines indicate the position of domain swaps to generate chimeras (see Supplementary Figure S3). **(B)** Serial tenfold dilutions of indicated strains were spotted on minimal media containing either glucose or oleic acid as sole carbon source. Plates were photographed after 2 days of incubation at 23°C. The assays reveal that both the NTD and the CTD of Pex5a and Pex5b contribute to peroxisome function to a different extent. It is yet unclear why growth of mutants only expressing the NTD of Pex5b is heavily affected on glucose media. **(C)** Fluorescence microscopic images of indicated strains expressing the peroxisomal marker protein mCherry-SKL. Scale bar represents 5 µm. Notably, the ∆*pex5a* *pex5b*:TPR_*pex5a*_ mutant shows peroxisomal localization of mCherry-SKL but is heavily affected in the growth assays (Figure 1B). **(D)** Serial tenfold dilutions of wild type cells, ∆*pex5a* mutants and ∆*pex5a* mutants overexpressing the NTD of Pex5a were spotted on minimal media containing either glucose or oleic acid as sole carbon source. Plates were photographed after 3 days of incubation at 23°C. Accordingly, Pex5a∆TPR is a functional targeting factor.

In the phytopathogenic fungus *U. maydis,* which causes smut disease on corn (Lanver et al., 2017), we have identified two Pex5-paralogs – termed Pex5a and Pex5b (Freitag et al., 2012). Pex5b is the longer paralog and contains a putative binding domain for the PTS2 receptor Pex7 (Figure 1A). Here, we show that the NTDs and the *C*-terminal TPR-domains of Pex5a and Pex5b each can recognize and import specific cargo. Additionally, we found that the NTD of Pex5b is essential for import of all peroxisomal matrix proteins investigated, and thus acts as a critical component in a dynamic network of receptors that target soluble proteins into peroxisomes.

## Results

### Functional Analysis of Pex5a and Pex5b From *U. maydis*

In a previous study we have described two Pex5-like proteins, Pex5a (Um02528) and Pex5b (Um10172) from *U. maydis* required for optimal growth of the fungus in different nutrient conditions and for pathogenic development (Figure 1A; Freitag et al., 2012). To discriminate the specific contributions of both proteins for growth on different carbon sources (Freitag et al., 2012; Camões et al., 2015), we tested *pex5a* and *pex5b* single and double deletion mutants on media containing either glucose or oleic acid as sole carbon source (Figure 1B). We found that ∆*pex5a* cells did not exhibit a severe growth defect on glucose plates but on oleic acid-containing medium (Figure 1B). Growth of mutants lacking *pex5b* or both genes was reduced on glucose-containing medium indicated by smaller colonies on solid medium and lower growth rates in liquid medium (Figure 1B and Supplementary Figure S1). On plates containing oleic acid as sole carbon source proliferation of these mutants was almost abolished (Figure 1B). This indicates that the presence of both Pex5 proteins is required for peroxisome function in *U. maydis*.

Next, we followed localization of the peroxisomal reporter protein mCherry-SKL in ∆*pex5a* and ∆*pex5b* cells (Figure 1C). Peroxisomal localization of mCherry-SKL was observed in ∆*pex5a* mutants but not in ∆*pex5b* mutants suggesting that Pex5b is necessary for peroxisomal import of PTS1 proteins in *U. maydis*. To test whether impaired binding of PTS1 cargo was responsible for the phenotype of ∆*pex5b* cells we deleted the TPR domains of Pex5b. Although the partial deletion of *pex5b* affected growth on oleic acid-containing medium (Figure 1B), *pex5b∆TPR* mutants still were able to import mCherry-SKL into peroxisomes (Figure 1C). Pex5b can therefore import PTS1 proteins into peroxisomes in the absence of Pex5a, while Pex5a-mediated protein import depends on the Pex5b NTD.

To address whether the observed growth phenotype of ∆*pex5a* cells (Figure 1B) results from different specificities of the Pex5a and Pex5b receptors for distinct subsets of peroxisomal matrix proteins or is caused by the reduced overall amount of Pex5 receptors, we overexpressed Pex5 derivatives. Overexpression of Pex5b rescued the growth phenotype of ∆*pex5b* cells but did not restore the growth phenotype of ∆*pex5a* cells (Supplementary Figures S2A,B). This indicates that it is not the reduced dosage of TPR proteins but rather the specificity of the Pex5a receptor, which explains the phenotype of ∆*pex5a* mutants.

We addressed this hypothesis by construction of strains expressing Pex5a and Pex5b chimeric variants (Supplementary Figure S3). A mutant expressing Pex5 proteins containing only the TPR domain of Pex5a (*pex5b:*TPR*_pex5a_*) exhibited a much stronger growth defect on oleic acid-containing medium compared to a mutant expressing only the TPR domain of Pex5b (*pex5a:*TPR*_pex5b_*), which only showed a slightly reduced colony size (Figure 1B). The TPR domain of Pex5b, therefore, recognizes specific PTS1 proteins required for peroxisome function in these conditions. This is further supported by the finding that, although growth was abolished on oleic acid-containing medium (Figure 1B), mCherry-SKL is localized to peroxisomes in ∆*pex5a pex5b:*TPR*_pex5a_* cells indicating that this chimeric protein is not generally affected in peroxisomal import of PTS1 proteins (Figure 1C).

In addition, our experiments reveal that the absence of the NTD of Pex5a is primarily responsible for the growth defect of the ∆*pex5a* strain on oleic acid-containing medium (Figure 1B, compare mutant ∆*pex5a* with *pex5a:TPR*_*pex5b*_). Indeed, overexpression of Pex5a∆TPR was able to rescue the phenotype of a ∆*pex5a* strain (Figure 1D). Critical peroxisomal matrix proteins are likely to exist, which specifically require the NTD for import. Moreover, the data suggest that Pex5a can act as a targeting factor even in the absence of its TPR domains.

### Identification of Pex5a-Specific Cargo

**FIGURE 2:**
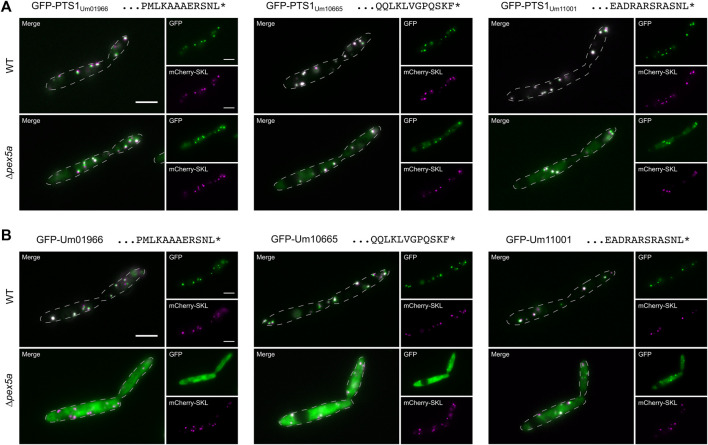
Identification of three cargo proteins of Pex5a. Fluorescence microscopic images of indicated strains expressing the peroxisomal marker protein mCherry-SKL (magenta) together with GFP (green) fused to *C*-terminal dodecamers including the PTS1 motifs of the *U. maydis* proteins Um01966, Um10665 and Um11001 **(A)**, with GFP-tagged full-length version of these proteins **(B)**. Scale bars represent 5 µm. These experiments show that localization of Um01966, Um10665 and Um11001 to peroxisomes is affected upon deletion of *pex5a*.

Several proteins from other fungi are known, which are imported into peroxisomes *via* binding to the NTD of Pex5 although they lack a canonical PTS or the PTS1 has been removed (van der Klei and Veenhuis, 2006). We reasoned that PTS1-containing substrates that require the NTD of Pex5a may as well display specificity for the TPR domain of this cargo receptor. To identify such proteins, we generated a library of GFP proteins with *C*-terminal dodecameric PTS1 motifs derived from *U. maydis* enzymes presumably involved in peroxisomal ß-oxidation (Table 1; Figure 2A and Supplementary Figure S4; Brocard and Hartig, 2006; Camões et al., 2015). Constructs were expressed in WT and in ∆*pex5a* cells (Supplementary Figure S4). GFP fused to PTS1 motifs of the candidate proteins Um01966, Um10665 and Um11001 showed peroxisomal localization in WT cells but accumulated in the cytosol in the absence of Pex5a (Figure 2A and Supplementary Figure S4).

**TABLE 1:** Candidates tested in the screen for Pex5a cargo.

Functional prediction	*U.MAYDIS* GENE	PTS1
Acyl-CoA oxidases	um01966	PMLKAAAERSNL*
	um02028	GEAVPFTERARL*
	um02208	TDFDSDLPRAKL*
Acyl-CoA dehydrogenases	um06400	ALLAKAGIKSHL*
	um01466	QALRMMPENARL*
	um00122	WTQGSGDVKSHL*
	um10665	QQLKLVGPQSKF*
Enoyl-CoA hydratases	um01747	VANDDVARFAKL*
	um02097	LAPPSSHARSKL*
	um11001	EADRARSRASNL*
	um10825	IRLDGASRLGKL*
Sterol carrier proteins	um11938	LDGVLKSQKAKL*
	um01850	NEVKKMSRVAKL*
Dienoyl-CoA isomerase	um01273	VMQKQTPKFAKL*
3,2-Trans-enoyl-CoA isomerases	um01599	FENIAAGARHKL*
	um03158	ESLRAAAAKSKL*

Um01966 is a putative acyl-CoA oxidase with high similarity to yeast Pox1, Um10665 a putative acyl-CoA dehydrogenase and Um11001 a putative enoyl-CoA hydratase (Table 1; Camões et al., 2015). *N*-terminally GFP-tagged full-length versions of all three proteins co-localized with mCherry-SKL in the presence of Pex5a, but showed pronounced cytosolic localization in ∆*pex5a* cells (Figure 2B). Cytosolic accumulation of GFP-tagged full-length proteins was even enhanced compared to the *C*-terminal dodecamers fused to GFP. Both experiments show that the three identified proteins are cargo with a preference for Pex5a.

### Combinatorial Interaction With the NTD and the TPR Domain of Pex5a Determines Import Specificity

**FIGURE 3:**
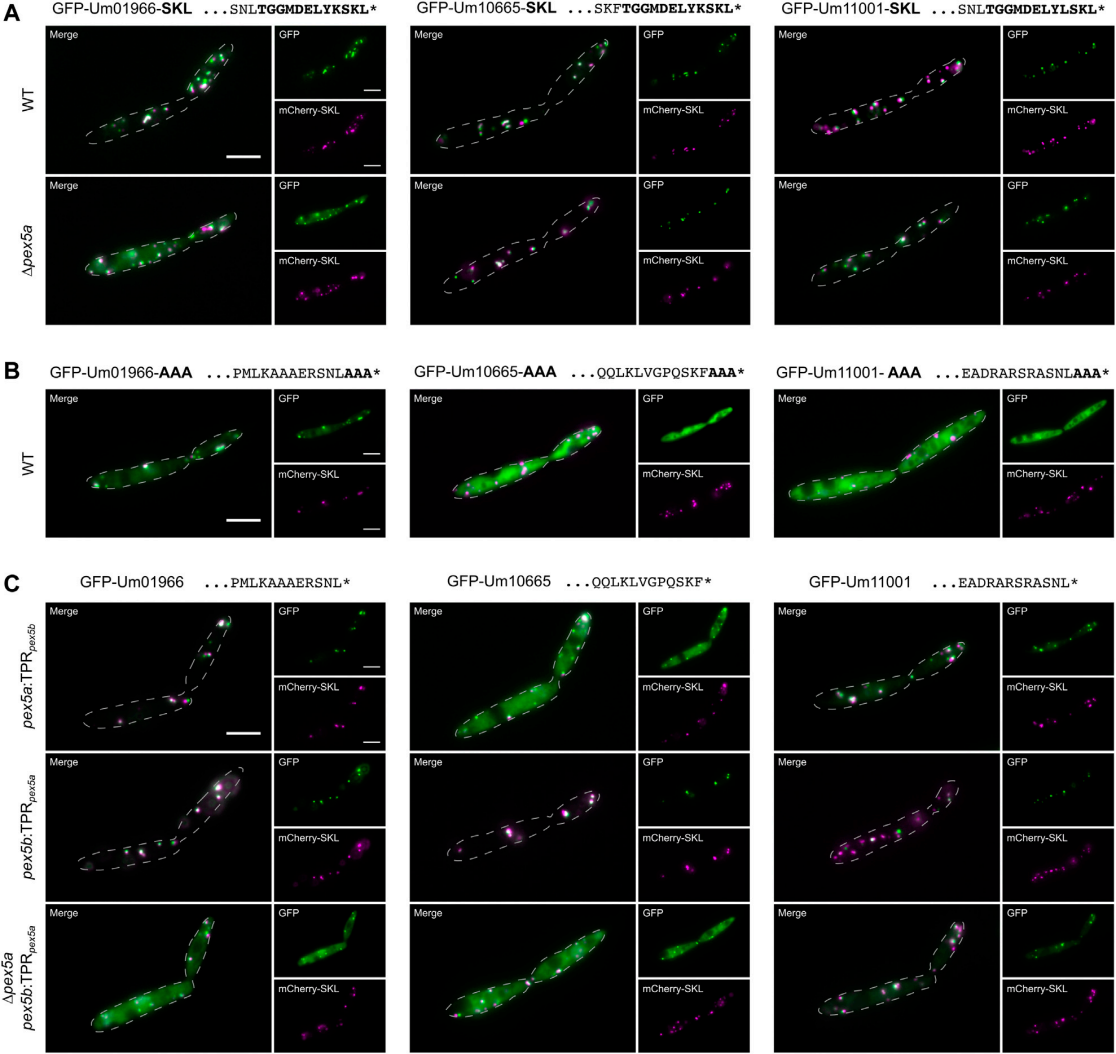
The NTD of Pex5a acts as targeting factor for Um01966. Fluorescence microscopic images of indicated strains expressing the peroxisomal marker protein mCherry-SKL (magenta) together with GFP-tagged full-length proteins (green) with their usual PTS1 masked by SKL **(A)** or AAA **(B)** in ∆*pex5a* and ∆*pex5b* cells. **(C)** Representative images of GFP-tagged full-length proteins expressed in mutants containing chimeric receptor proteins. These data reveal that both the NTD and the CTD contribute to cargo import.

To discriminate between the function of the *C*-terminal TPR domain and the NTD of Pex5a for peroxisomal import of Um01966, Um10665 and Um11001 we added canonical SKL containing motifs at the *C*-terminus of each. Targeting to peroxisomes in ∆*pex5a* cells was drastically improved for GFP-Um10665-SKL and GFP-Um11001-SKL. Um01966-SKL predominantly co-localized with mCherry-SKL positive foci in wild type cells but substantial cytosolic mistargeting was observed in a strain deleted for *pex5a* (Figure 3A). This suggests a critical function of Pex5a for import of the acyl-CoA oxidase Um01966, which cannot be entirely bypassed by addition of a *C*-terminal canonical targeting signal.

Upon masking the PTS1 by *C*-terminal addition of three alanine residues (AAA), GFP-Um10665-AAA and GFP-Um11001-AAA remained largely cytosolic, while GFP-Um01966-AAA partially co-localized with mCherry-SKL (Figure 3B). A GFP-tagged and AAA-masked *C*-terminal dodecamer of Um01966 remained cytosolic revealing an additional targeting signal within the full-length protein (Supplementary Figure S5). Microscopic analysis of strains expressing chimeric variants of Pex5a and Pex5b demonstrated that the Pex5a TPRs are not required for efficient targeting of GFP-Um01966 to peroxisomes (Figure 3C). However, in the absence of the NTD of Pex5a (∆*pex5a pex5b:TPR*_*pex5a*_) residual peroxisomal targeting of GFP-Um01966 was observed (Figure 3C), while GFP-Um01966 localized almost entirely in the cytosol upon depletion of *pex5a* (Figure 2B). This is probably due to the presence of the Pex5a TPRs, which might partially compensate for the absence of the Pex5a NTD in ∆*pex5a pex5b:TPR*_*pex5a*_ cells (Figure 3C). These results are in accordance with our initial observation that the isolated PTS1 containing sequence requires Pex5a for efficient import (Figure 2A). Targeting of GFP-Um10665 and to a minor extent GFP-Um11001 was reduced in the absence of each part of Pex5a (Figure 3C). Together, these experiments suggest that it is the combination of affinities to the NTD and the CTD of Pex5a, which facilitates recognition and import of Pex5a specific cargo. The impact of each of the two domains can be different depending on individual substrates.

### PTS1 Motifs With Higher Affinity to the TPR Domains of Pex5a

**FIGURE 4:**
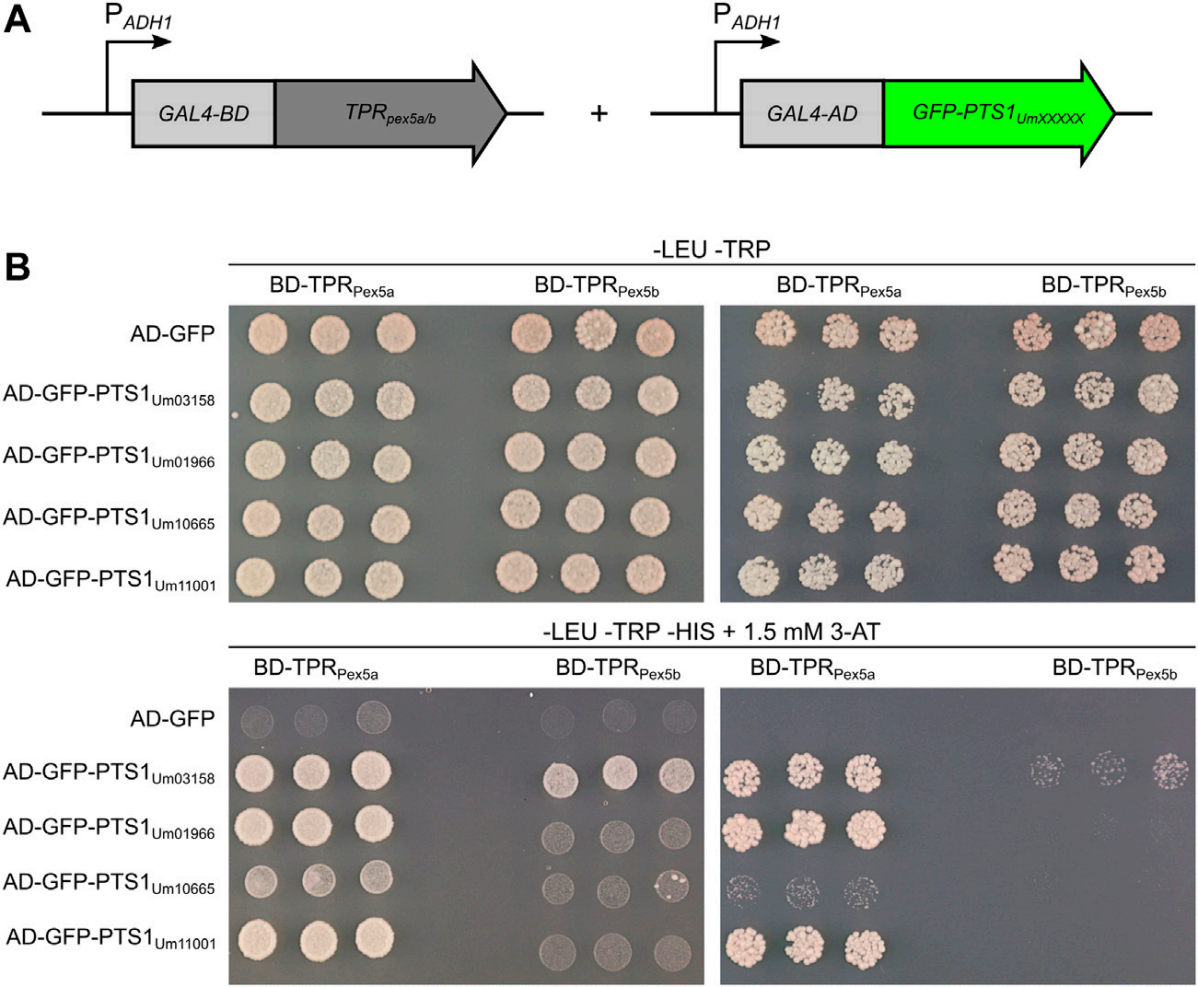
PTS1 motifs with higher affinity to Pex5a. **(A)** Schematic drawing of constructs used for the yeast two-hybrid assay. Constructs were expressed in a strain derived from AH109 deleted for *PEX5* (Stehlik et al., 2020). TPR domains of either Pex5a or Pex5b were fused to the GAL4 DNA-binding domain (GAL4-BD), while *C*-terminal dodecamers of candidate proteins were fused to the GAL4 activation domain (GAL4-AD). **(B)** Fivefold and fiftyfold dilutions (OD_600_ = 0.2 and 0.02, respectively) of three independent transformants co-expressing indicated constructs were spotted on -LEU -TRP media and -LEU -TRP -HIS media and incubated for 3 days at 30°C. The selection medium (-LEU -TRP -HIS) contained 1.5 mM 3-Amino-1,2,4-triazole. This assay demonstrates that Um01966, Um10665 and Um11001 are substrates of the Pex5a TPRs and show only a very weak interaction with the Pex5b TPRs.

To test the binding affinities of TPR domains of Pex5a and Pex5b (Figure 2), we set up a yeast two-hybrid experiment (Chien et al., 1991). We attached the TPR domains of either protein to the Gal4-DNA-binding domain (BD), and GFP with *C*-terminal dodecamers of different candidate proteins to the Gal4-activation domain (AD) (Figure 4A). As a control we used a *C*-terminal dodecamer, which efficiently mediates import of GFP into peroxisomes in the absence of Pex5a (Supplementary Figure S3; Um03158). Constructs were transformed in respective combinations into AH109∆*pex5* (Stehlik et al., 2020). Interactions were monitored for three independent transformants per combination by growth assays on high stringency medium (Figure 4B). The assay revealed a stronger interaction of PTS1 motifs of Um01966, Um10665 and Um11001 with the TPR domain of Pex5a compared to the TPR domain of Pex5b. Among the tested candidates the PTS1 motif of Um03158 had the highest affinity to the TPRs of Pex5b and may thus be efficiently imported *via* both Pex5 proteins (Figure 4B). Interaction data from the two-hybrid experiment are in agreement with the data on import efficiency obtained by fluorescence microscopy (Figure 2). The weak interaction of the Um10665 PTS1 with the Pex5a TPRs may explain why efficient import of GFP-Um10665 is only observed when a native Pex5a protein containing the original NTD and CTD is expressed (Figure 3C). The strong interaction of the PTS1 of Um01966 with the Pex5a TPRs explains residual peroxisomal import of GFP-Um01966 in ∆*pex5a pex5b:TPR*_*pex5a*_ cells (Figure 3C)*.* Together, the results of the two-hybrid assay confirm that TPR domains of Pex5a and Pex5b show distinct preferences for variations of the *C*-terminal targeting signal and indicate that both cargo receptors have specific as well as shared substrates.

### PTS1 Motifs With a Preference for Pex5b

**FIGURE 5:**
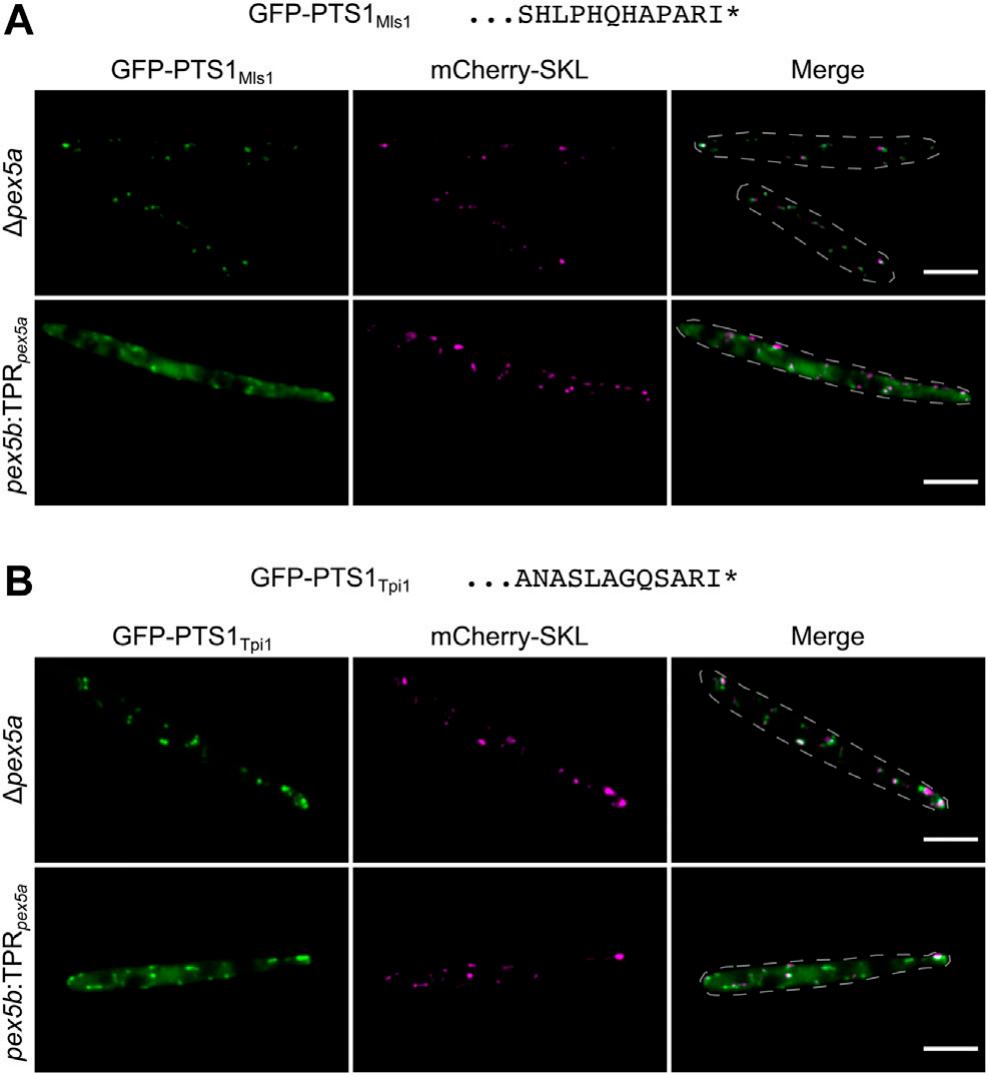
PTS1 motifs with a preference for Pex5b TPRs. Fluorescence microscopic images of indicated strains expressing the peroxisomal marker protein mCherry-SKL (magenta) together with GFP (green) fused to *C*-terminal dodecamers of Mls1 (malate synthase) **(A)** and Tpi1 (triosephosphate isomerase) **(B)**. Both *C*-terminal dodecamers end on identical tripeptides and show efficient peroxisomal import when the TPR domains of Pex5b were present. Scale bars represent 5 µm.

Import of the putative Pox1 ortholog Um01966 shows similarity to Pox1 in *S. cerevisiae* since both can be imported *via* the NTD of a Pex5 protein (Klein et al., 2002). We wondered whether more similarities exist and tested import specificity of the glyoxylate cycle enzyme Mls1 – a Pex9 substrate in *S. cerevisiae* (Effelsberg et al., 2016; Yifrach et al., 2016) – in cells containing Pex5 derivatives with only one type of TPRs. To this end we fused the *C*-terminal dodecamers of the *U. maydis* malate synthase ortholog Mls1 (Um15004) to GFP. Indeed, we detected efficient peroxisomal import in the presence of the TPR domain of Pex5b but pronounced cytosolic localization when only the TPR domain of Pex5a was present (Figure 5A). mCherry-SKL predominantly localized in peroxisomes of both strains (Figure 5A). Mls1 from *U. maydis* contains the *C*-terminal tripeptide ARI. Remarkably, also a stop codon readthrough derived isoform of the glycolytic/gluconeogenetic enzyme triosephosphate isomerase (Tpi1; Um03299) harbors this *C*-terminal tripeptide (Freitag et al., 2012) and efficient import into peroxisomes also depends on the TPRs of Pex5b (Figure 5B). Thus, the highly similar PTS1 motifs of both enzymes are preferentially recognized by Pex5b.

### Pex5b as Key Player for Matrix Protein Import in *U. maydis*

Of the cytosolic receptors, only Pex5b can mediate peroxisomal import as a single protein, while Pex5a requires the NTD of Pex5b (Figure 1). Pex5b might act as a co-receptor for Pex5a similar to Pex5-large for Pex7 in mammals or Pex18 and Pex21 for Pex7 in *S. cerevisiae* (Braverman et al., 1998; Otera et al., 1998; Purdue et al., 1998; Woodward and Bartel, 2005). Alternatively, Pex5b could be independently required for the activity of the peroxisomal import machinery.

**FIGURE 6:**
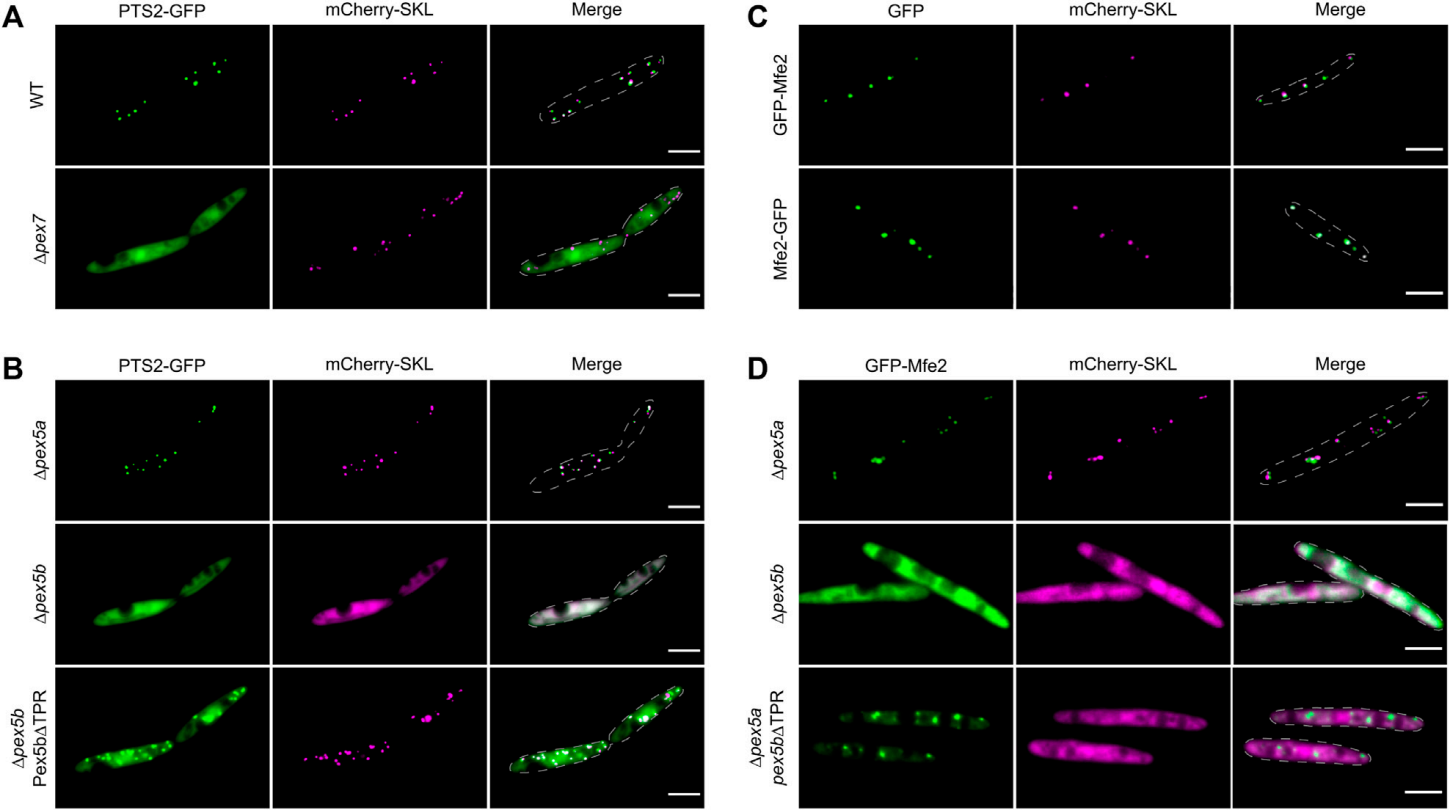
The NTD of Pex5b is a crucial factor for matrix protein import in *U. maydis*. Fluorescence microscopic images of indicated strains expressing mCherry-SKL (magenta) together with PTS2-GFP (green) **(A**,**B)** or with GFP-tagged Mfe2 (green) **(C**,**D)**. The data show that the NTD of Pex5b is involved in peroxisomal targeting of PTS2 proteins and more unusual cargo such as the multifunctional enzymes (Mfe2). Scale bars represent 5 µm.

In *U. maydis* PTS2-dependent transport may also involve Pex5b as a co-receptor, since the NTD of Pex5b contains a putative binding site for Pex7 (Figure 1; Kiel et al., 2006). In addition, the *U. maydis* genome lacks any ortholog of the yeast co-receptors Pex18 and Pex21 (Kämper et al., 2006). To test Pex5b for targeting of PTS2 proteins, we engineered a reporter protein by fusing an *N*-terminal fragment of the PTS2 protein Um01090 to GFP (PTS2-GFP; Figure 5A). The *N*-terminus of this predicted 3-keto-acyl-CoA thiolase related to yeast Pot1 contains a sequence matching the PTS2 consensus [R/K]-[L/V/I]-[X]5-[H/Q]-[L/A] (Lazarow, 2006; Camões et al., 2015; Kunze, 2020). We observed co-localization of PTS2-GFP with mCherry-SKL in peroxisomes and found that PTS2-GFP was retained in the cytosol upon deletion of the *pex7* ortholog (*um03596*) (Figure 6A)*.* To address whether Pex5b acts as co-receptor for Pex7, we expressed PTS2-GFP in *pex5a* and in *pex5b* deletion mutants. While peroxisomal localization was not affected in ∆*pex5a* cells, absence of *pex5b* completely abolished peroxisomal import of PTS2-GFP (Figure 6B). Reintroduction of the NTD of Pex5b into ∆*pex5b* mutants partially restored targeting of PTS2-GFP to peroxisomes suggesting that the NTD of Pex5b acts as co-receptor for Pex7 in *U. maydis* (Figure 6B).

### Pex5b-Dependent Import of a Matrix Protein Without a Canonical PTS

Finally, we focused on peroxisomal matrix proteins without a canonical peroxisomal targeting signal. Several proteins have been described that neither contain a PTS1 nor a PTS2 but, nevertheless, localize inside of peroxisomes (van der Klei and Veenhuis, 2006). In *U. maydis*, the multifunctional enzyme involved in peroxisomal fatty acid oxidation (Mfe2; Um10038) was characterized earlier (Klose and Kronstad, 2006). Although we could not detect any PTS motif in Mfe2, both *N*-terminally and *C*-terminally tagged GFP fusion proteins co-localized with mCherry-SKL suggesting that Mfe2 is imported into peroxisomes independently of recognition by Pex7 or the Pex5 TPRs (Figure 6C). We probed the mode of transport of Mfe2 by localization studies in a set of *U. maydis* mutants and found that peroxisomal targeting of Mfe2 requires Pex5b. The NTD of Pex5b was sufficient for partial peroxisomal localization of GFP-Mfe2 but import of Mfe2 was more efficient when the full-length protein was expressed (Figures 6C,D). This shows that the NTD of Pex5b also can act as receptor for peroxisomal matrix proteins in *U. maydis*.

## Discussion

**FIGURE 7:**
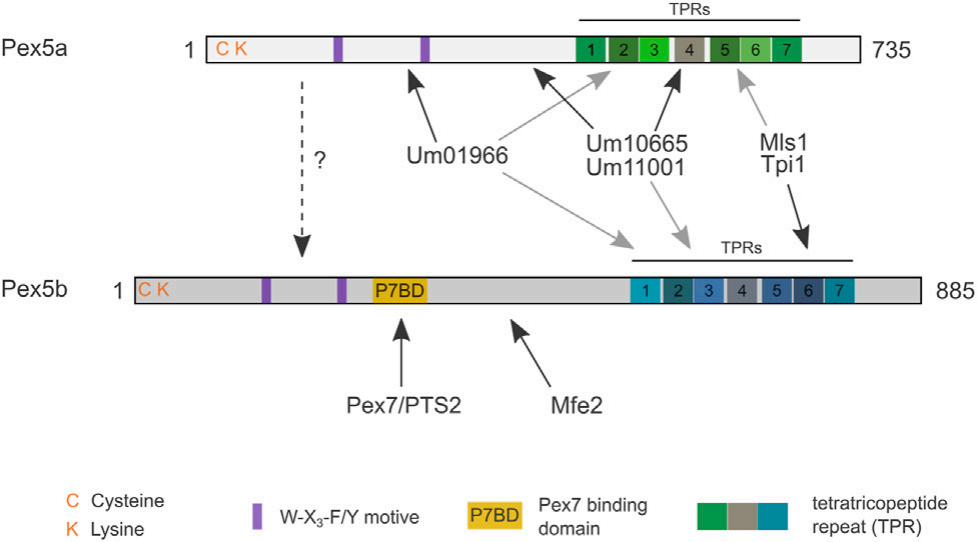
Model highlighting preferential cargo of the different domains of Pex5a and Pex5b. Cargo proteins of different domains are shown. Black arrows point to preferred binding domains. Gray arrows point to secondary binding sites. Please note that the cargo affinities of the TPRs are overlapping, while PTS2 import *via* Pex7 seems to be a specific function of Pex5b. For Mls1 and Tpi1 only specificity of the *C*-terminal dodecamer was determined. If an interaction between Pex5a and Pex5b can occur during import (dashed arrow) or if other functions of Pex5b determine its critical role for Pex5a-dependent import remains to be established.

Our work uncovered a network of import modules for peroxisomal matrix proteins in *U. maydis* (Figure 7). We have identified five modules: Pex5b can transport substrates destined for the peroxisomal matrix either *via* its NTD (as coreceptor for Pex7 bound to PTS2-GFP, and Mfe2) or *via* its TPR domain (direct recognition of PTS1-containing proteins). The TPR domains of Pex5a and Pex5b bind to PTS1 motifs with overlapping but not identical specificity (Figures 2–5). The NTD of Pex5a also contributes to targeting but does not facilitate peroxisomal import in the absence of Pex5b (Figures 1–3, 7). Although the NTD of Pex5a is shorter in comparison to Pex5b, it contains the evolutionary conserved di-aromatic motifs for interaction with Pex14 (Figure 1). It is currently unclear why Pex5a alone is not sufficient to mediate cargo import. Recently, a role of Pex5 for insertion of membrane proteins was observed in *S. cerevisiae* (Martenson et al., 2020). A similar function of the Pex5b NTD may indirectly contribute to the critical importance of Pex5b for matrix protein import in *U. maydis* e.g., by targeting membrane proteins of the importomer.

Our genetic analyses demonstrate that two paralogs of Pex5 are necessary for optimal growth on oleic acid medium. Interestingly, key enzymes of the β-oxidation pathway seem to be preferentially targeted to peroxisomes *via* different factors. Pex5a is responsible for targeting of the acyl-CoA oxidase Um01966 related to *S. cerevisiae* Pox1, the multifunctional enzyme Mfe2 is imported into peroxisomes in the absence of Pex5a while the thiolase Um01090 depends on Pex7 for peroxisomal import (Figures 2, 6). Three different import routes may ensure the correct stoichiometry of enzymes inside of each peroxisome in particular when peroxisomes proliferate. Of interest, the *S. cerevisiae* ortholog Pox1 can bind to the NTD of Pex5 to target peroxisomes (Skoneczny and Lazarow, 1998; Klein et al., 2002). Malate synthase belongs to the cargo recognized by the Pex5 paralog Pex9 in *S. cerevisae* (Effelsberg et al., 2016; Yifrach et al., 2016; Yifrach et al., 2022). In *U. maydis* Mls1 is a preferred substrate of Pex5b. Thus, an evolutionary conservation of targeting mechanisms for particular peroxisomal proteins may exist indicating biological relevance of specific routes for specific enzymes.

In addition, we observed a growth defect on glucose medium for several of the *pex5* mutants. A similar phenotype was already detected for *U. maydis* ∆*pex3* and ∆*pex6* cells as well as in other fungi (Idnurm et al., 2007; Freitag et al., 2012; Camões et al., 2015). Previously, we identified a metabolic network of carbohydrate metabolizing enzymes that are dually targeted to peroxisomes and the cytosol in a number of fungi (Freitag et al., 2012; Stiebler et al., 2014; Freitag et al., 2018; Kremp et al., 2020)*.* A function of peroxisomes in regulating gluconeogenesis was recently described for *S. cerevisiae* and a number of novel, often dually localized peroxisomal proteins was discovered (Yifrach et al., 2021). Many metabolic and regulatory functions of peroxisomes still await elucidation and may contribute to reduced fitness observed for peroxisome-deficient mutants in glucose-containing medium. It is yet obscure, why growth of a mutant expressing only the NTD of Pex5b was more affected than any of the other strains (Figure 1). A possible explanation might be that import of only a subset of proteins e.g. Mfe2 or Pot1 is more detrimental for cells than retention of all peroxisomal matrix proteins in the cytosol.

Several peroxisomal proteins without a canonical PTS such as Mfe2 are known to bind to the NTD of Pex5 in *S. cerevisiae* (Skoneczny and Lazarow, 1998; Klein et al., 2002; Rymer et al., 2018; Kempiński et al., 2020). Aox1, Cta1, Fox2, Pox1 and Pox4 from different yeast species (Small et al., 1988; Kragler et al., 1993; Skoneczny and Lazarow, 1998; Gunkel et al., 2004; Rymer et al., 2018) resemble Um01966 and can be imported into peroxisomes if the PTS1 motifs are removed pointing to additional targeting signals. More such proteins likely exist, but two targeting signals may appear redundant and are therefore hard to detect. Specificity for a Pex5 protein seems to result from the combination of affinities towards the NTD and the CTD – for different cargo interaction with one or the other domain is more relevant or may even be sufficient (Figures 2–6). A very recent study focusing on TPR domains of the paralogs Pex5 and Pex9 from *S. cerevisiae* revealed that substrates can be discriminated by amino acids in vicinity of the *C*-terminal tripeptide (Yifrach et al., 2022). If this also applies to *U. maydis* or if other features of the motif determine specificity is an exciting question for future research. The *C*-terminal tripeptide could be important as well, as two of the three identified Pex5a substrates end on SNL, while two identified cargoes of Pex5b contain the tripeptide ARI at the *C*-terminus (Figures 2, 5).

Pex7-mediated import in *U. maydis* differs from several yeast species since specific co- receptors for PTS2 import are not present (Purdue et al., 1998; Schäfer et al., 2004; Kiel et al., 2006). We could show that in *U. maydis* PTS2 import depends on the NTD of Pex5b and this pathway shows more similarity to plants and mammals (Braverman et al., 1998; Otera et al., 1998; Woodward and Bartel, 2005; Kunze et al., 2015).

Allosteric interactions upon cargo binding are important for turning Pex5 and Pex7 into import-competent receptors attaching to the docking complex followed by translocation and cargo release (Kunze et al., 2015; Bürgi et al., 2021). The Pex5 CTD inhibits docking of the NTD in the absence of cargo; the NTD can translocate into the peroxisomal membrane when the CTD is deleted (Klein et al., 2002; Gouveia et al., 2003; Gunkel et al., 2004; Dias et al., 2017). In agreement with these data we found that overexpression of Pex5a lacking the TPRs can rescue the growth defect of ∆*pex5a* cells (Figure 1). Furthermore, we detected targeting of GFP-Mfe2 and PTS2-GFP to peroxisomes upon expression of the Pex5b NTD in the absence of the full-length protein (Figure 5). The capability of the peroxisomal import machinery to translocate large oligomeric cargo has been described (Walton et al., 1995; Yang et al., 2018). It will be interesting to establish how interactions at different sites of Pex5 proteins influence import of bigger complexes and import kinetics. In addition, the exact mechanistic function of both Pex5 proteins might be worth to evaluate.

Taken together, our study reveals the impact of different domains of Pex5 paralogs on cargo recognition and on peroxisome function in different growth conditions and contributes to a better understanding of peroxisomal protein import. Versatile import routes for matrix proteins seem to be widespread and may be critical for the formation of functional peroxisomes in many species.

## Methods

### Generation of Strains and Nucleic Acid Procedures

Constructs were generated using standard cloning procedures (Sambrook et al., 1989) or Gibson assembly (Gibson et al., 2009). All plasmids were verified by sequencing. *Escherichia coli* strain Top Ten (Invitrogen) was used for transformation according to a standard protocol (Hanahan et al., 1991) and propagation of plasmid DNA. Transformation of *U. maydis* cells was achieved as described previously (Schulz et al., 1990). Deletion strains and chimeric variants were created using an *Sfi1* based cloning system (Brachmann et al., 2004; Kämper, 2004; Supplementary Figure S3). Genomic DNA was extracted according to an established protocol (Hoffman and Winston, 1987). Mutant strains were verified by Southern blot analysis (Sambrook et al., 1989). Proteins were expressed under control of the constitutive *otef*-promoter either integrated into the *cbx-*locus (Broomfield and Hargreaves, 1992; Spellig et al., 1996) or randomly integrated into the genome of *U. maydis* (mCherry-SKL)*.* Similar expression levels were confirmed by measuring fluorescence using Synergy Mx multimode microplate reader (BioTek). All plasmids, strains and oligonucleotides used or generated during this study are listed in Supplementary Table S1. Progenitor plasmids used for this study were described (Spellig et al., 1996; Brachmann et al., 2004; Sandrock et al., 2006; Freitag et al., 2012; Stehlik et al., 2020). Genes can be accessed on NCBI.

### Growth Conditions

*U. maydis* strains were grown at 28°C in liquid YEPSL (1% yeast extract, 0.4% peptone, 0.4% sucrose) or on solid potato dextrose broth containing 1.5% Bacto agar at 28°C. For selection solid media were supplemented with antibiotics (Brachmann et al., 2004). For growth assays 4 µl of serial tenfold dilutions of logarithmically growing cells starting with an OD_600_ of 1 (Figure 1B and Supplementary Figure S2) or 0.1 (Figure 1D) were spotted on solid minimal yeast nitrogen based media (Difco) with a pH of 5.7 supplemented with 0.5% ammonium sulfate. The plates contained 1.5% Bacto agar and either 2% glucose or a mixture of 0.2% oleic acid and 0.05% Tween-40. Plates were incubated for two (Figure 1B and Supplementary Figure S2) to 3 days (Figure 1D) at 23°C. All assays were at least repeated three times with similar results.

### Growth Assays in Liquid Media

Cells of an OD_600_ of 1 were diluted to a starting OD_600_ of 0.1 and inoculated into fresh yeast nitrogen based media (Difco) with a pH of 5.7 supplemented with 0.5% ammonium sulfate and 2% glucose in a volume of 100 µl in flat bottom 96 well plates. Growth assays were performed in a Synergy Mx multimode microplate reader (BioTek) at 23°C with high shaking setting. OD_600_ was determined in 30 min intervals. Each strain was measured in five technical replicates and in three independent experiments. Mean values were plotted and original data and standard deviations are accessible in Supplementary Table S2.

### Microscopy

A total of 200 µl of hot 1.5% agarose melted in water was used to create a thin agarose cushion on a 76 × 26 mm microscope slide (Roth). Cells from logarithmic growth phase incubated in yeast nitrogen based media (Difco) with a pH of 5.7 supplemented with 0.5% ammonium sulfate and 2% glucose were washed with water, concentrated fivefold, and 3 µl were spotted onto the middle of the agarose pad and covered with an 18 × 18 mm coverslip (Roth). Microscopy was performed on an Axiovert 200 M inverse microscope (Zeiss) equipped with a 1394 ORCA-ERA-CCD camera (Hamamatsu Photonics), filter sets for enhanced GFP (EGFP) and rhodamine (Chroma Technology), and a Zeiss 63×Plan Apochromat oil lens (NA 1.4). Single-plane bright field or phase contrast images and z-stacks of the cells (0.5 µm z-spacing) in the appropriate fluorescence channels were recorded, using the image acquisition software Volocity 5.3 (Perkin-Elmer). Images were processed and evaluated in ImageJ (Schneider et al., 2012). For protein localization analysis, single plain images or *z*-projections of deconvolved image stacks were used. Deconvolution was performed on the *z*-stacks by the ImageJ plugin DeconvolutionLab with 25 iterations of the Richardson – Lucy algorithm (Sage et al., 2017).

### Yeast Two-Hybrid Assay

Sequences encoding the TPR domains of Pex5a and Pex5b were inserted into pGBKT7 (Matchmaker GAL4 Two-Hybrid System 3; Clontech) between the EcoRI and BamHI restriction sites *via* Gibson assembly. The ORFs for GFP or GFP modified with *C*-terminal dodecamers of Um01966, Um10665, Um11001, and Um03158 including PTS1 motifs were cloned into pGADT7 (Matchmaker GAL4 Two-Hybrid System 3; Clontech) between the EcoRI and BamHI restriction sites. Either pGBKT7-Pex5aTPR or pGBKT7-Pex5bTPR were co-transformed with one of the pGADT7 plasmids into YTS398, a derivative AH109 of deleted for *pex5* (Stehlik et al., 2020). Three independent transformants of each of the 10 combinations were grown in liquid synthetic defined (SD) medium lacking leucine and tryptophan to an OD_600_ of approx. 1. Cells were washed once with sterile water and 4 µl of fivefold or fiftyfold dilutions (OD_600_ = 0.2 or 0.02) were spotted on solid SD medium lacking leucine and tryptophan as growth control, and on SD medium lacking leucine, tryptophan and histidine, and containing 1.5 mM 3-amino-1,2,4-triazole to test for protein – protein interaction. Plates were incubated for 3 days at 30°C.

## Data Availability

The original contributions presented in the study are included in the article/Supplementary Material, further inquiries can be directed to the corresponding author.
