# Supplementary material for: Two Pex5 Proteins With Different Cargo Specificity Are Critical for Peroxisome Function in Ustilago maydis
Source: Front Cell Dev Biol. 2022 May 12;10:858084. doi: 10.3389/fcell.2022.858084 (PMC9133605; doi:10.3389/fcell.2022.858084)
Supplement: Supplementary file 2 [file Presentation1.pdf]

Supplementary figures

Ast et al., 2022

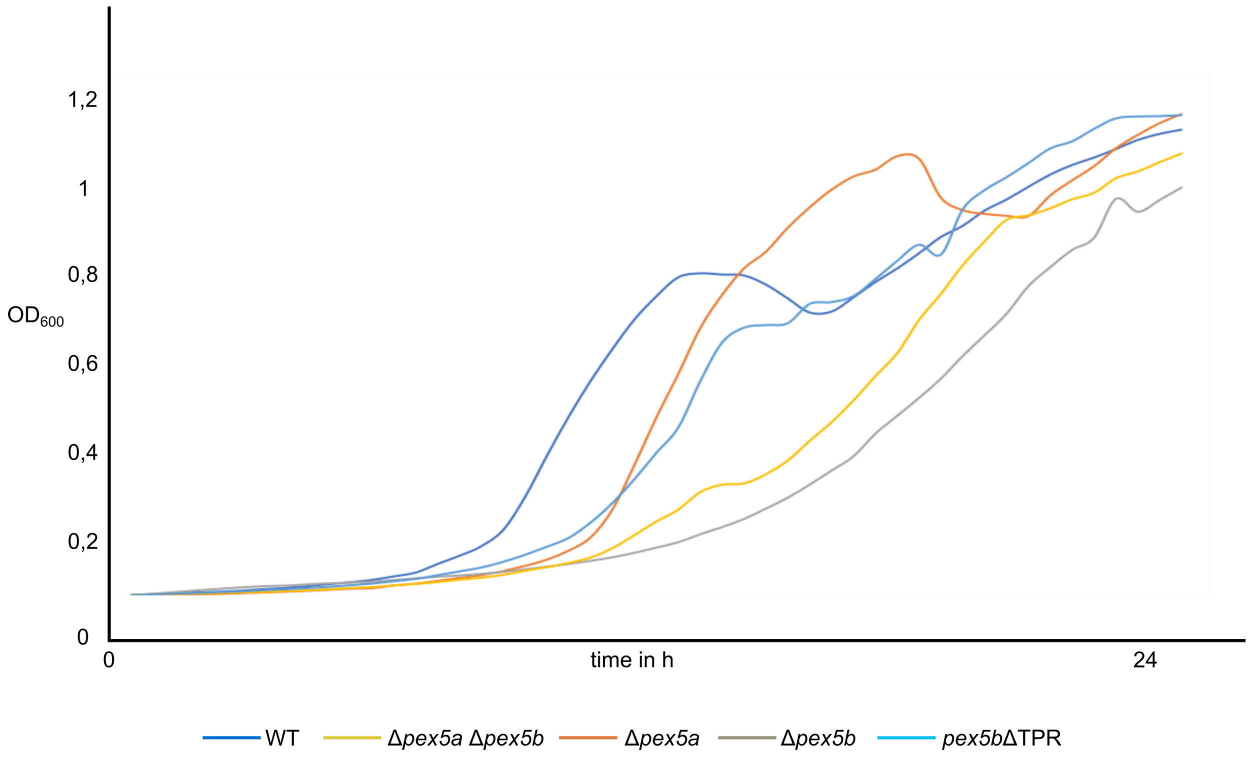

Figure S1: Growth assay in glucose containing liquid medium

Logarithmically growing cells were incubated in microtiter plates in a BioTek microplate reader at 23°C using high shaking conditions. OD_{600} was measured every 30 min. Shown are the mean values of three independent experiments each based on five technical replicates. Original data and standard deviations are accessible in table S2. The assays revealed a stronger growth defect of Δ*pex5b* and Δ*pex5a*Δ*pex5b* mutants compared to WT cells and mutants only lacking Pex5a or the TPR domain of Pex5b. Please note that the intermediate decrease for some of the strains was not further followed up. It may result from morphological changes of the fungus, but does not interfere with the interpretation of growth rates in logarithmic phase.

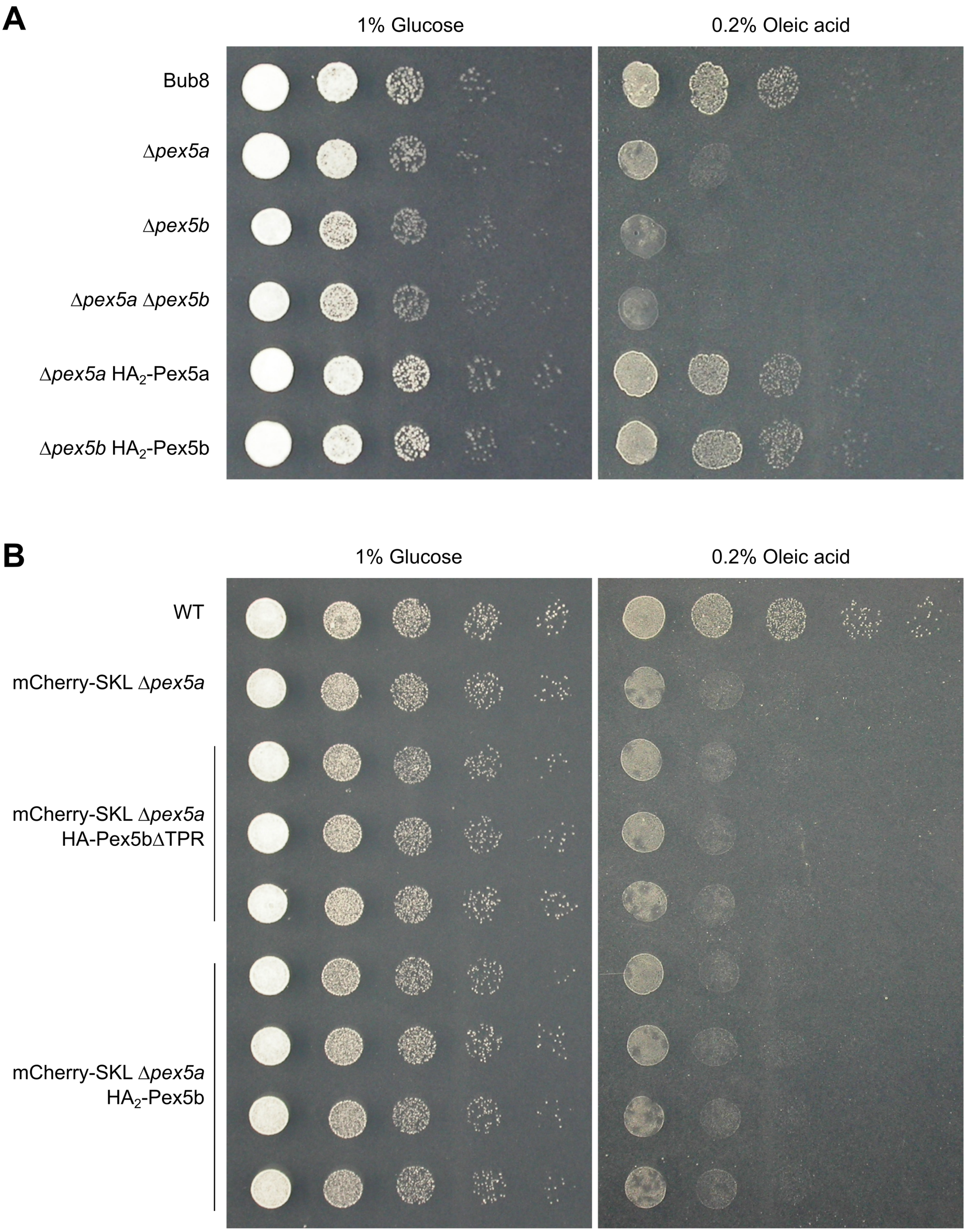

Figure S2: Overexpression of Pex5b does not suppress the growth defect of cells lacking Pex5a. (A) Serial tenfold dilutions of indicated strains were spotted on minimal media containing either glucose or oleic acid as sole carbon source. Derivatives of HA-tagged Pex5 proteins were expressed under control of the constitutive *otef* promoter (Spellig *et al.,* 1996). (B) The experiment was performed as in (A). These data suggest that it is not the amount of a Pex5 proteins but rather the cargo selectivity responsible for the phenotype of Δ*pex5a* cells.

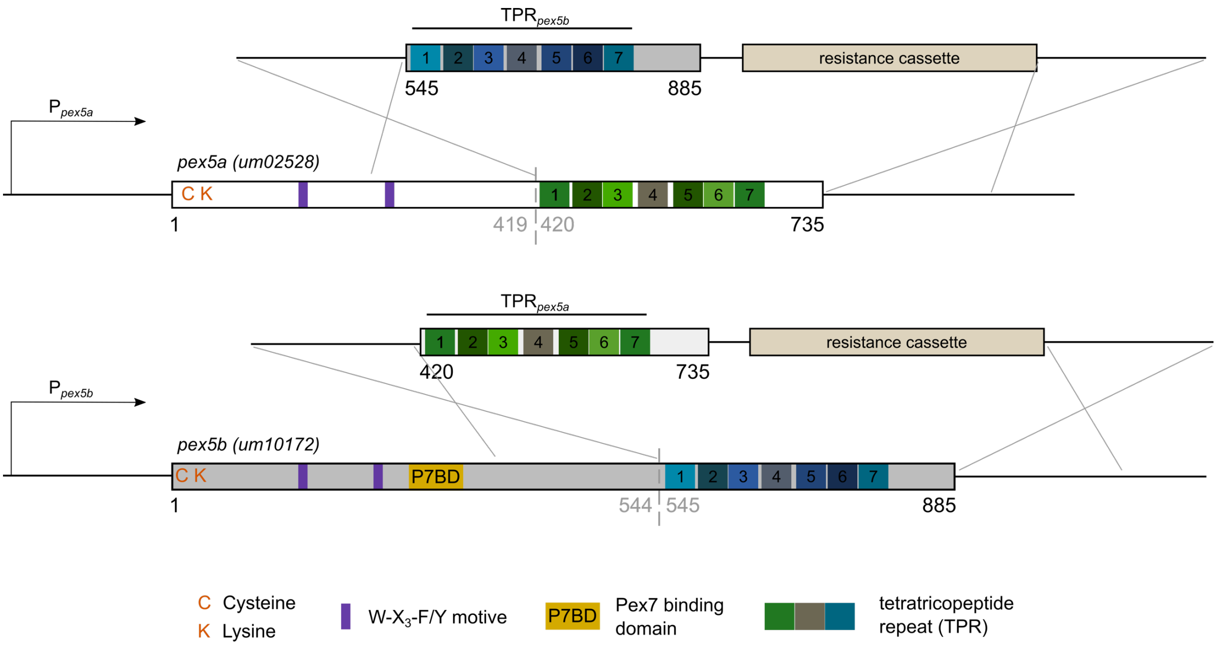

Figure S3: Scheme highlighting the construction of chimeric versions of Pex5a and Pex5b

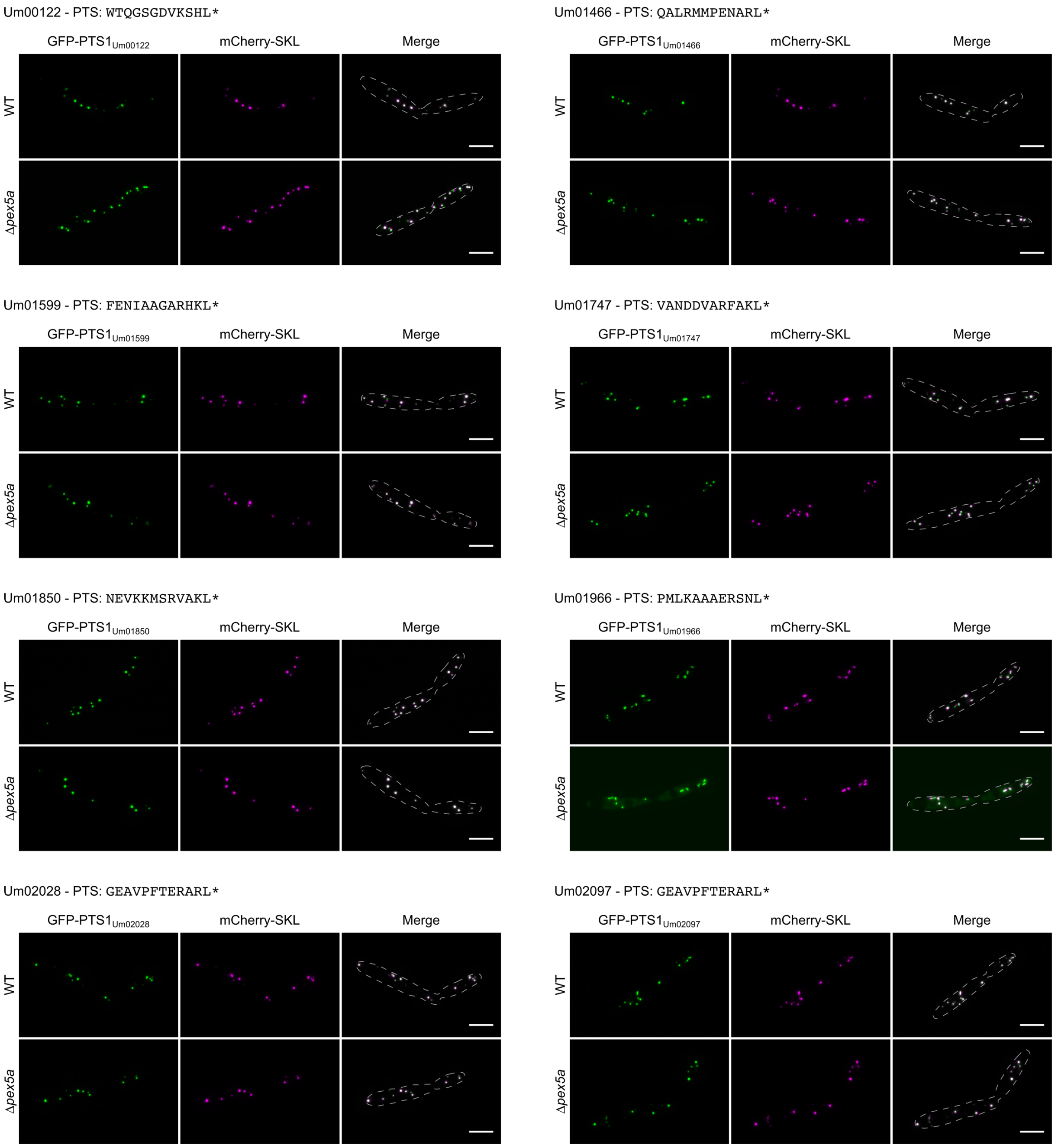
Um00122 - PTS: WTQGSGDVKSHL*
GFP-PTS1$_{Um00122}$
mCherry-SKL
Merge
WT
Δpex5a
Um01466 - PTS: QALRMMPENARL*
GFP-PTS1$_{Um01466}$
mCherry-SKL
Merge
WT
Δpex5a
Um01599 - PTS: FENIAAGARHKL*
GFP-PTS1$_{Um01599}$
mCherry-SKL
Merge
WT
Δpex5a
Um01747 - PTS: VANDDVARFAKL*
GFP-PTS1$_{Um01747}$
mCherry-SKL
Merge
WT
Δpex5a
Um01850 - PTS: NEVKKMSRVAKL*
GFP-PTS1$_{Um01850}$
mCherry-SKL
Merge
WT
Δpex5a
Um01966 - PTS: PMLKAAAERSNL*
GFP-PTS1$_{Um01966}$
mCherry-SKL
Merge
WT
Δpex5a
Um02028 - PTS: GEAVPFTERARL*
GFP-PTS1$_{Um02028}$
mCherry-SKL
Merge
WT
Δpex5a
Um02097 - PTS: GEAVPFTERARL*
GFP-PTS1$_{Um02097}$
mCherry-SKL
Merge
WT
Δpex5a

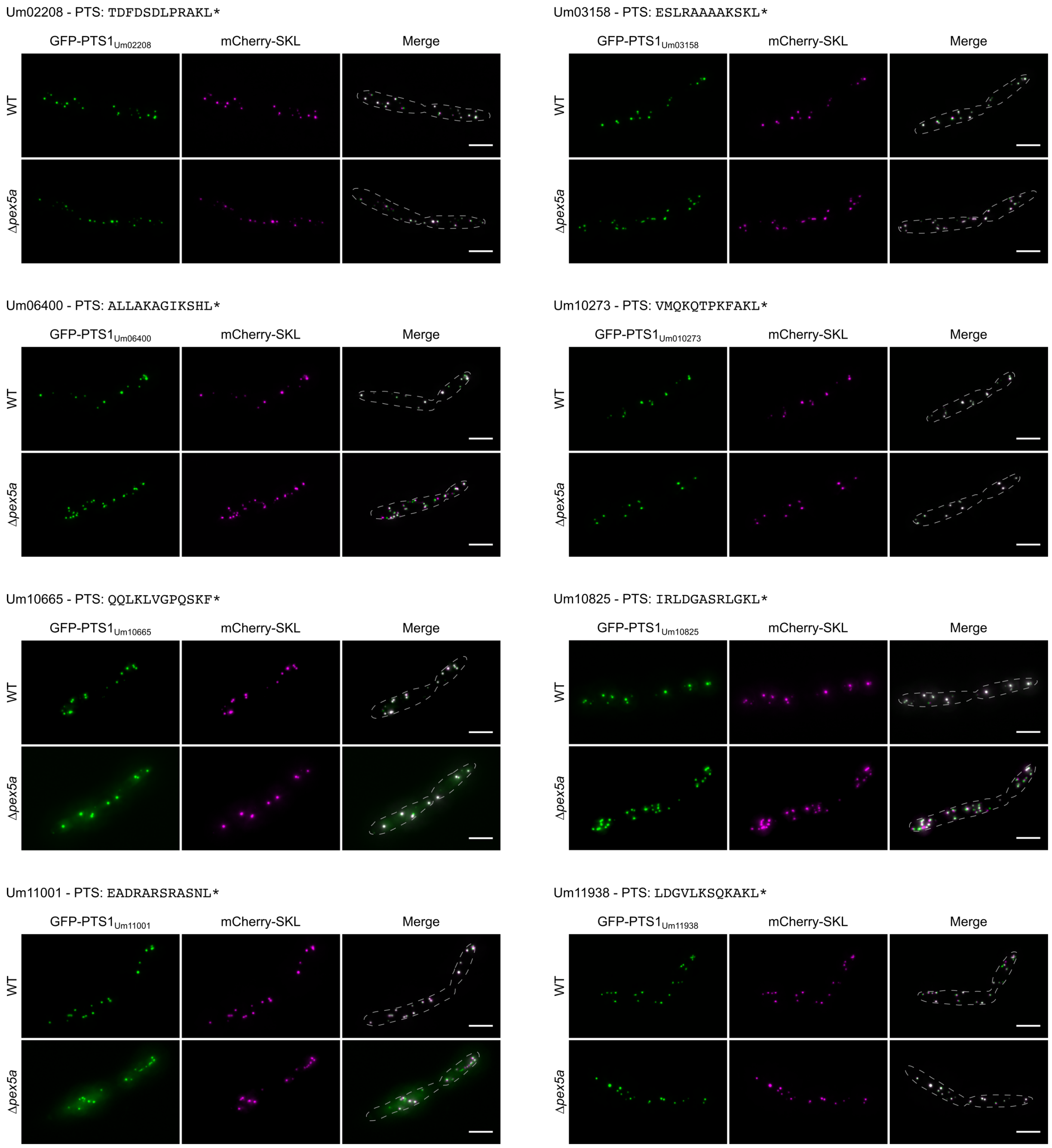

Figure S4: Screen for Pex5a cargo.

C-terminal dodecamers derived from enzymes potentially involved in peroxisomal β-oxidation (Tab. 1) were fused to GFP and tested for peroxisomal localization in wildtype and Δ*pex5a* cells. mCherry-SKL served as peroxisomal marker protein. Scale bars represent 5 µm. Three PTS1 containing sequences were identified, which led to reduced import of GFP in Δ*pex5a* cells compared to wild type cells (Um01966; Um10665; Um11001).

GFP-PTS1$_{Um01966-AAA}$ ...PMLKAAAERSNLAAA*

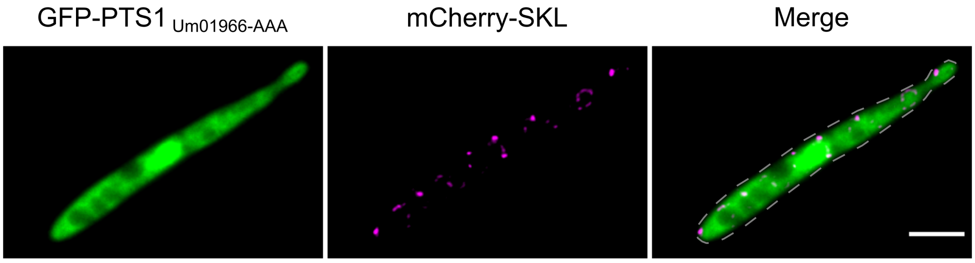

Figure S5: Masking the C-terminal dodecamer of Um01966 with three alanine residues.
Fluorescence microscopic images of a cell expressing GFP (green) with the masked PTS1 of Um01966 ending on AAA together with the peroxisomal marker protein mCherry-SKL (magenta). Scale bar represents 5 µm.
